# Diseases spectrum in the field of spatiotemporal patterns mining of infectious diseases epidemics: A bibliometric and content analysis

**DOI:** 10.3389/fpubh.2022.1089418

**Published:** 2023-01-09

**Authors:** Weili Lu, Hongyan Ren

**Affiliations:** ^1^State Key Laboratory of Resources and Environmental Information System, Institute of Geographic Sciences and Natural Resources Research, Chinese Academy of Sciences, Beijing, China; ^2^College of Resources and Environment, University of Chinese Academy of Sciences, Beijing, China

**Keywords:** spatiotemporal patterns, bibliometrics, content analysis, diseases spectrum, research directions, research gaps, infectious diseases

## Abstract

Numerous investigations of the spatiotemporal patterns of infectious disease epidemics, their potential influences, and their driving mechanisms have greatly contributed to effective interventions in the recent years of increasing pandemic situations. However, systematic reviews of the spatiotemporal patterns of communicable diseases are rare. Using bibliometric analysis, combined with content analysis, this study aimed to summarize the number of publications and trends, the spectrum of infectious diseases, major research directions and data-methodological-theoretical characteristics, and academic communities in this field. Based on 851 relevant publications from the Web of Science core database, from January 1991 to September 2021, the study found that the increasing number of publications and the changes in the disease spectrum have been accompanied by serious outbreaks and pandemics over the past 30 years. Owing to the current pandemic of new, infectious diseases (e.g., COVID-19) and the ravages of old infectious diseases (e.g., dengue and influenza), illustrated by the disease spectrum, the number of publications in this field would continue to rise. Three logically rigorous research directions—the detection of spatiotemporal patterns, identification of potential influencing factors, and risk prediction and simulation—support the research paradigm framework in this field. The role of human mobility in the transmission of insect-borne infectious diseases (e.g., dengue) and scale effects must be extensively studied in the future. Developed countries, such as the USA and England, have stronger leadership in the field. Therefore, much more effort must be made by developing countries, such as China, to improve their contribution and role in international academic collaborations.

## 1. Introduction

Infectious diseases (ID), a leading cause of morbidity and mortality worldwide, place a huge burden on the world, particularly in certain countries, regions, and territories, along with increasingly changing climate conditions and globalization (1, 2). It is important to understand the spatiotemporal patterns through various mining methods, and the complex process of disease transmission, its potential influences, and its driving mechanisms. This would provide insightful guidance to health authorities for making and implementing targeted interventions for infectious diseases.

Previous studies have pointed out that the spatiotemporal patterns of infectious disease prevalence are a macroscopic result of the interaction between the virus/pathogens, vectors, and hosts (3). Spatiotemporal patterns refer to the spatiotemporal distribution characteristics of geographical objects, including frequency (frequent paths, mainly for moving objects), aggregation, periodicity (periodic and seasonal characteristics of research objects), correlation (changes in research objects over time), and co-location (frequent spatial proximity between objects) (4, 5). Thus, mining infectious disease spread and transmission patterns on various spatial scales and temporal intervals would detect the diseases' epidemiological characteristics, explore their potential influences, clarify their influence mechanisms, predict epidemic risks under various conditions, based on the spread and transmission simulation, and evaluate the feasibility or effectiveness of preventive and control measures implemented by relevant health authorities (5, 6). In other words, this paradigm covers nearly every aspect of infectious diseases, except for the isolation or laboratory testing of the pathogens, through which effective interventions for various infectious diseases were achieved in the past decades (5, 6).

Due to successful performance in other research fields, a series of spatiotemporal analytical methods or models, such as kernel density analysis, spatial-time scan statistics, geo-detector, and random forest, have been appropriately employed for mining the spatial and temporal patterns of various communicable diseases (7–22). Meanwhile, different data sources and acquisitions were required for completing spatiotemporal pattern mining since the spread and transmission were commonly caused by different viruses or pathogens through specific vectors or direct human-to-human transmission. They included gene sequence data for infectious diseases from public databases, vector sentinel monitoring data, and infectious disease cases data based on streaming or reporting that covered pathogens, vectors, hosts, and environmental suitability (pathogens' survival and transmissibility, vectors' breeding, and hosts' being infected) (3). In addition, the rising utility of individual activity information, derived from social media or other mobility data, provided opportunities for clarifying the role of individuals in disease transmission and assessing the risk of being infected (23). The question remains, since the results and related conclusions were achieved on a certain fine scale (i.e., relative microscopic level), were the containment measures and interventions, based on these microscopic findings, appropriately implemented at mesoscale or macroscale? In addition, some other concerns in this field include hot diseases and their spectrum and the academic leadership of international collaborations network among countries, institutes, or scholars, which have seldom been focused on in previous literatures.

As efficient tools for reviewing literature, based on numerous publications, bibliometrics and content analysis have been widely applied in various fields to quantitatively explore the number of publications and their variations, arrange the concerned aspects and their timelines, and summarize the progress or proper prospects (24–27). These could provide useful clues and unique insights for scholars and health authorities. For example, Su et al. (28) summarized the disciplines involved, the main contributing countries, the main research themes, and the shortcomings of the existing research in the application of big data in carbon emissions and environmental management research based on bibliometric methods. Agnusdei and Coluccia (27) used bibliometrics and content analysis to summarize the trends and prospects in sustainable agri-food supply chain research, and four research themes were identified and several research gaps were discussed. Therefore, bibliometrics and content analysis were employed in this study to investigate the counts of publications regarding spatiotemporal patterns, dominant research directions of mining these patterns, the spectrum of infectious diseases concentrated upon in these studies, and the present scenario of international leadership and academic collaboration networks among countries.

## 2. Materials and methods

### 2.1. Sources of publications

Web of Science (WoS), the most frequently selected database for bibliometric studies in public health, was used for data retrieval in this study (25, 29, 30). The WoS is a comprehensive academic resource information platform established by the Institute for Scientific Information (ISI), one of the most comprehensive and influential mainstream databases in the world, including the Science Citation Index (SCI) database, which contains more than 9,000 high-quality academic journals in more than 170 disciplines, and the Social Sciences Citation Index (SSCI) database, with more than 3,000 authoritative academic journals in more than 50 social science disciplines. It provides scholars worldwide with detailed information about academic publications (29, 31).

### 2.2. Framework of search strategy, validation, and cleaning

**Figure 1:**
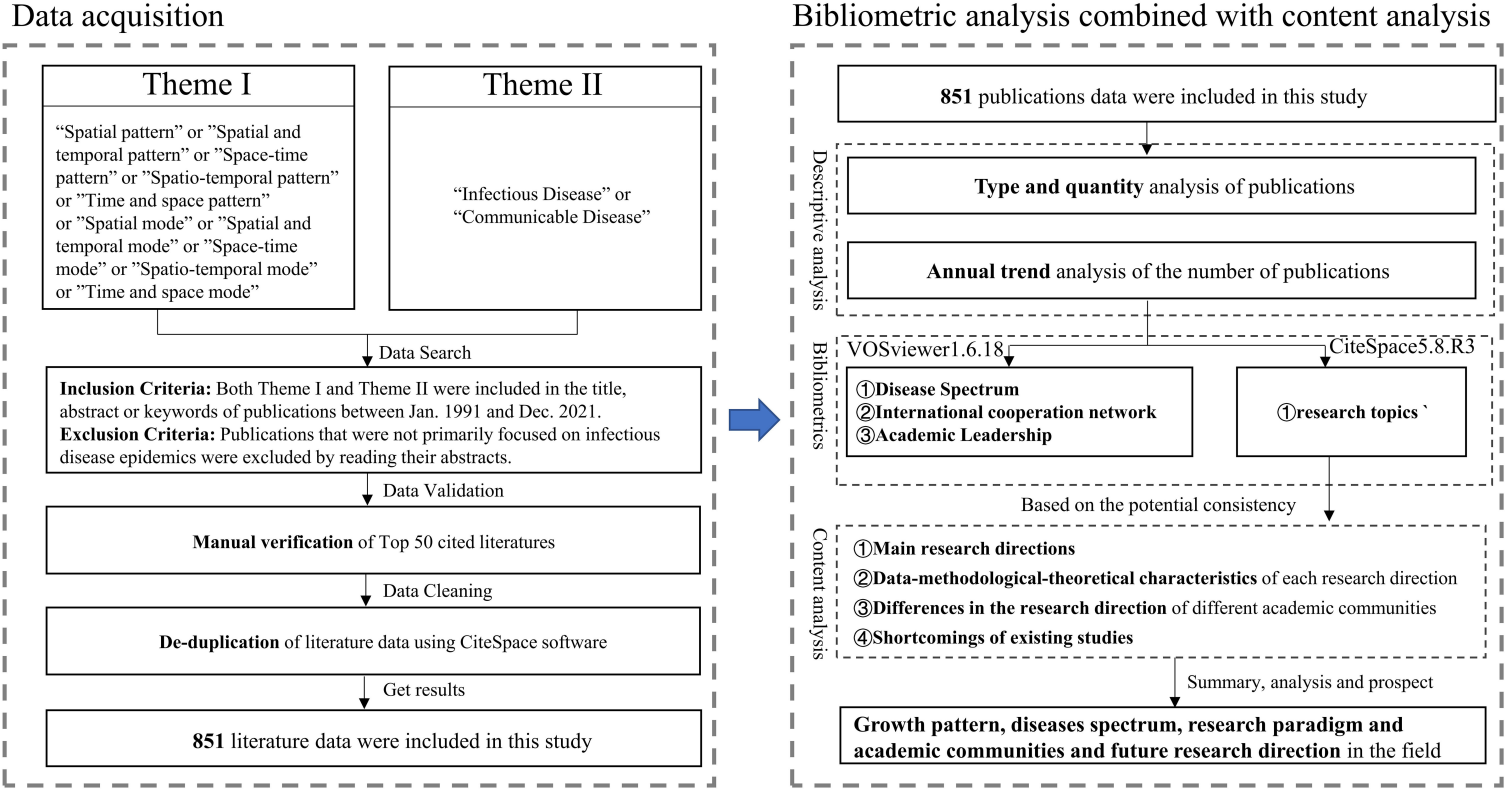
Research flowchart.

This study finalized a broad and comprehensive search strategy (Figure 1) after iterative testing to cover as many publications as possible while minimizing the number of irrelevant documents. Firstly, publications involving two themes (I for infectious diseases-related and II for spatiotemporal pattern-related) in the abstracts, titles, and keywords were selected as the basic dataset. Secondly, some publications in this dataset were then excluded while they factually emphasized on other topics, even though infectious disease epidemics were mentioned in their abstracts. For example, an article just referred to infectious disease patterns in the abstract, but it focused on the impacts of global warming on human society. A common validation method was also used to examine the search results to ensure that they met the scope of the research (25). The top 50 frequently cited publications in the search results were reviewed. All the reviewed publications met this study's scope of interest. A total of 851 publications were obtained after validation and deduplication, based on the above search strategy (the search was executed on January 3, 2022).

### 2.3. CiteSpace analysis

Bibliometric analysis has played an increasingly important role in the summary and outlook of several research fields (25, 29). As one of the most widely used tools CiteSpace, is known for its powerful literature co-citation analysis, which can mine research hotspots and grasp the research direction based on the references of the retrieved literature (32–35). The Log-Likehood Rate (LLR) algorithm in CiteSpace 5.8 R3 software was used for the co-citation analysis. It can cluster titles, abstracts, and keywords of publications to analyze and summarize the research themes in this study.

### 2.4. VOSviewer analysis

VOSviewer (36) is another frequently used bibliometric tool, popular for its accurate, clear, and detailed statistics and visualization functions. In this study, the software and statistical tools provided by the WoS website were used to conduct author keyword analysis, country cooperation network analysis, research institution analysis, and author-cited analysis to summarize and conclude the spectrum of infectious diseases, including academic cooperation and leadership in the field to analyze the current status of research in the field (Figure 1).

### 2.5. Content analysis

Although the research themes obtained by CiteSpace analysis were more objective, they were divided by infectious disease categories, rather than summarizing the macroscopic research (for the whole field) directions. Therefore, this study grouped the obtained research themes into several research directions based on the publication content under each theme. This method of analysis, based on the specific content of various types of research, is known to scholars as “content analysis” (37). The method summarizes the common spatiotemporal scales, data sources, and methods for each research direction and the least developed (blind spots) themes in the literature (to be read in light of the bibliometric results), allowing future research direction recommendations (27).

## 3. Result

### 3.1. Descriptive information of selected publications

As illustrated in Table 1, there were 851 publications derived from the core WoS database, including research articles (807), reviews (36), conference papers (17), and <10 miscellaneous items (e.g., editorials, book chapters, letters, etc.). As of January 3, 2022, the retrieved publications were cited 34,214 times (33,542 without self-citations) with an average of 40.2 citations per item. The frequency of citations illustrates that they possessed good representativeness.

**Table 1:** Types of included publications.

**Document types**	**Numbers**	**Percentage (%)**
Research articles	807	92.4
Review articles	36	4.1
Proceedings papers	17	1.9
Editorial materials	7	0.8
Book chapters	2	0.2
Early access	1	0.1
Letters	1	0.1
**Total**	**851**	**100**

The results of this category classification are provided by WoS. Since the same document may belong to two or more publication types at the same time, the sum of the numbers in the second column is greater than the total number of research publications.

**Figure 2:**
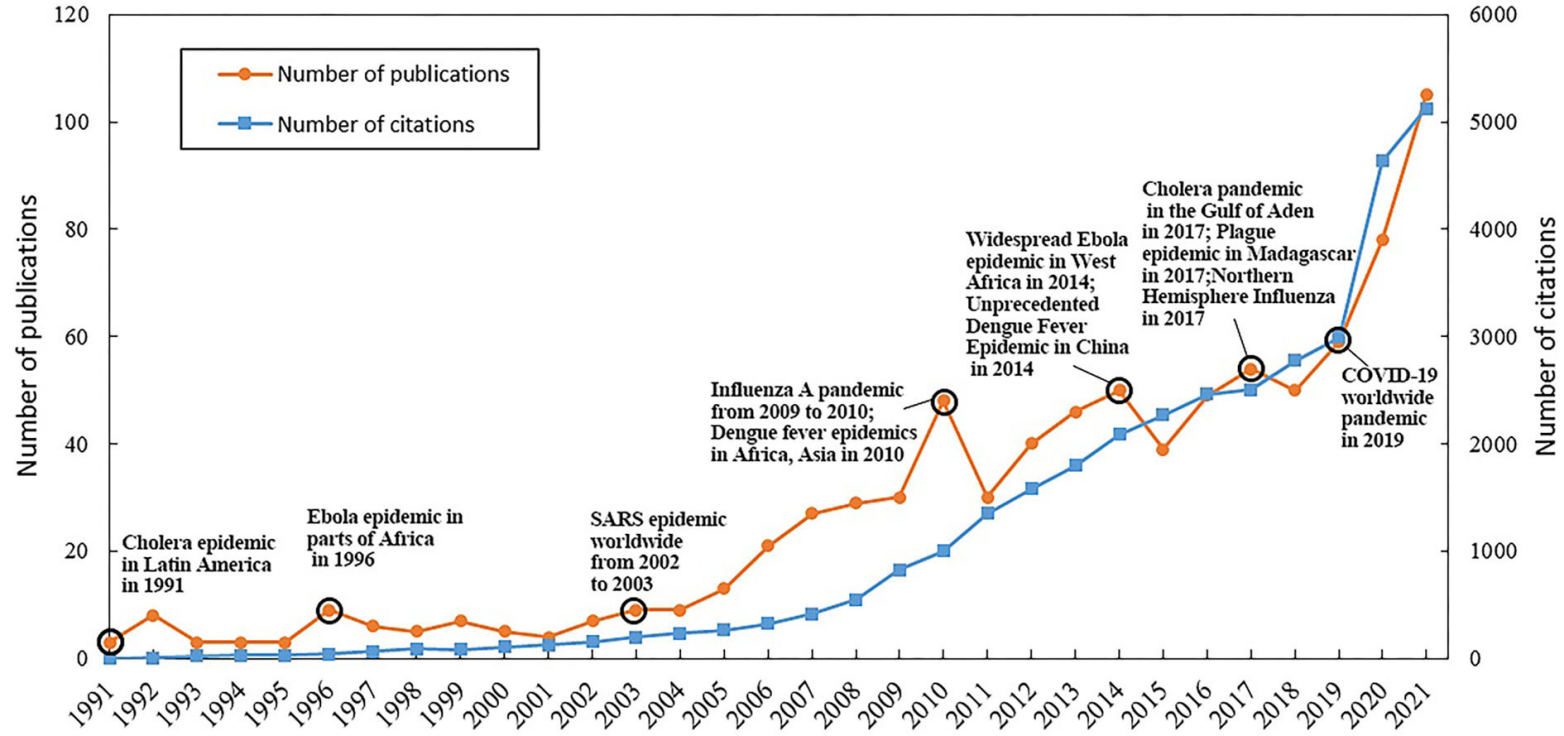
Annual change in the number of publications and citations.

At the same time, the number of included publications presents an upward trend over the past 30 years (Figure 2). Although there were three extreme years (2010, 2014, and 2017), the growth was divided into three periods based on the annual number of publications, including slow (1991–2003, <10 publications), fast (2004–2018, 10–50 publications), and rapidly rising (2019–2021, more than 50 publications). The significant phase's characteristics and extreme points of the annual publication amount curve may be driven by the outbreaks and epidemics of multiple infectious diseases.

### 3.2. Spectrum of infectious diseases

**Figure 3:**
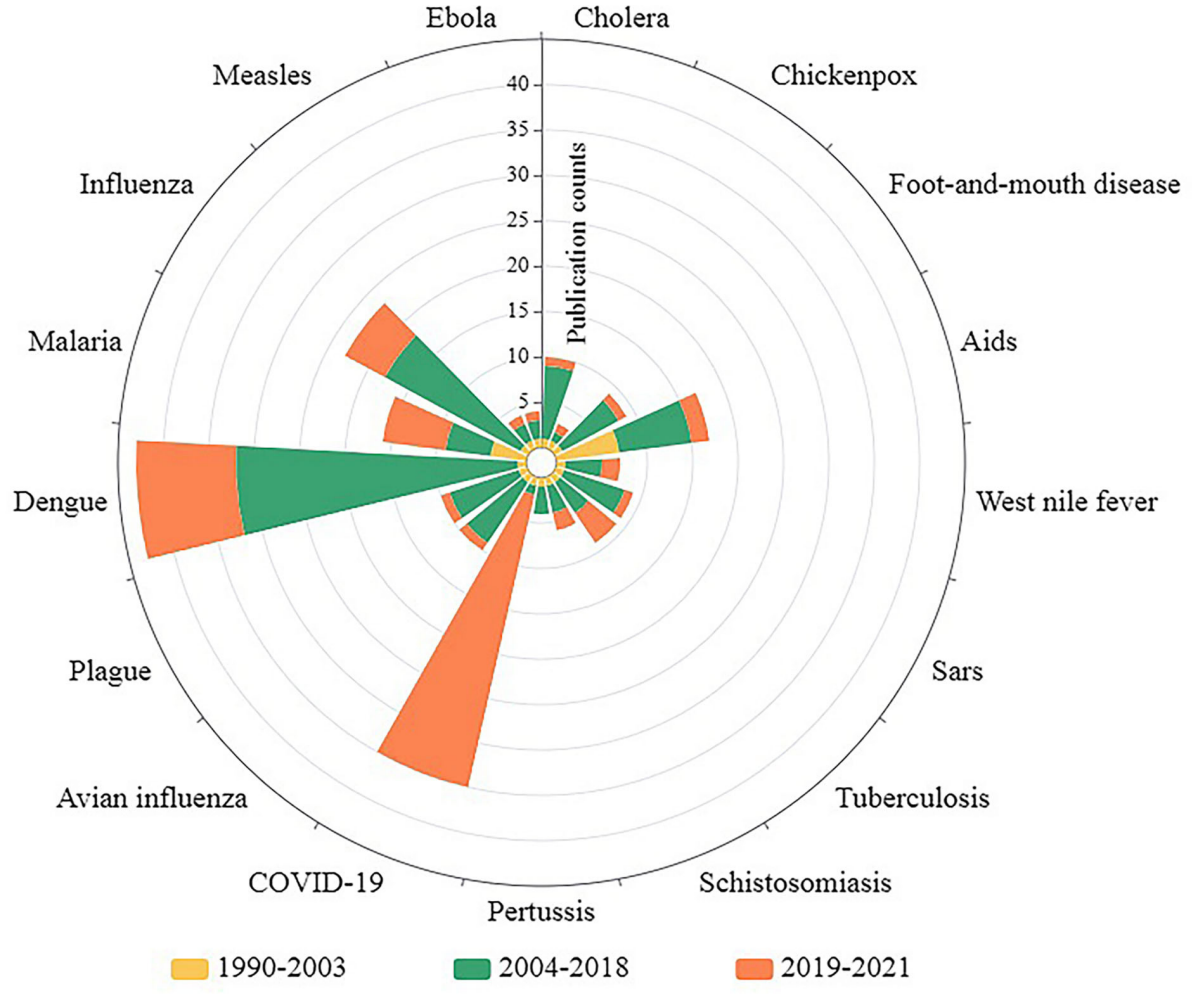
Related publication counts of main infectious diseases by publication stage.

The spectrum of infectious diseases studied in this field has changed significantly over the last three decades (Figure 3). A total of 13 typical infectious diseases were identified at the time (occurrences ≥5) of investigation in the above 851 publications. In particular, dengue (2004–2018, 2019–2021), COVID-19 (2019–2021), influenza (2004–2018, 2019–2021), AIDS (1990–2003, 2003–2018), and malaria (2004–2018, 2019–2021) were of major concern several times (more than five times in a single phase; more than 10 times in total) in one or multiple phases. Moreover, the prevalence of these diseases varied across the three periods. Certain infectious diseases (e.g., dengue in the fast-rising phase and COVID-19 in the rapid-rising phase) reached a high number of relevant publications (occurrences ≥30) at one stage, even though the previous stages were widely unnoticed (occurrences <5). This may be related to the sudden pandemic. While some infectious diseases, such as SARS, avian influenza, cholera, plague, and hand, foot, and mouth disease, were widely noted in the previous phases, related publications significantly declined in the new phase (2019–2021). This may be because the epidemics were effectively controlled. Nevertheless, common infectious diseases, such as dengue, COVID-19, influenza, and malaria, remain a widespread cause of concern in the latest stage (2019–2021) and deserve to be studied in depth.

### 3.3. Major research directions of selected publications

Nine research themes were identified in these publications through CiteSpace 5.8 R3 software. Each theme was based on distinctive data sources or applied research methods, focusing on the scientific questions in the context of specific diseases and their spatiotemporal patterns of prevalence (Supplementary Table S1). Three research directions (RD) were obtained according to their potential consistency in the aforementioned themes by content analysis (Table 2):

RD_1) Detecting spatiotemporal patterns (themes 1, 4, 8, and 11).
(RD_2) Exploring potential influencing factors of IDs' spatiotemporal patterns (themes 0, 2, 4, 7, and 11).
(RD_3) Simulating and predicting epidemic risk and evaluating prevention and control measures (themes 4, 5, 8, 9, and 11).

**Table 2:** Research directions information.

**Direction**	**Main concerns**				
	**Data source**	Methods (*)	**Spatiotemporal scale**	**Main pattern**	
Detection (RD_1)	Center for Disease Control and Prevention, WHO, Health commission, NHSO, Public Health Science Data Center, Statistical Yearbook, Published articles/datasets, Field trips and so on.	Space-time scan (72), Moran' I (63), Time series method (35), Spatiotemporal clustering (21), Getis-Ord Gi* (19), Probability distribution models (17), K function (17), Kernel density (13), Knox (10), Standard deviation ellipse (5)	Points, Administrative divisions (commune, district, city, province, country, etc.), Grids	Patterns of population distribution (age, gender, occupation, etc.), aggregation, periodicity. [e.g., the prevalence of dengue presented a multi-annual cycle of around 2–3-years in Vietnam due to climatic and entomological changes (50). The prevalence of tuberculosis had a particular population distribution pattern (51)].	
	**Data source**	Methods (*)	**Spatiotemporal scale**	**Factors (**+**)**	**Driving mechanism**
Exploration (RD_2)	WHO, World Bank, OSM, NASA, Geospatial Data Cloud, WMO, NOAA, WorldPop, Published articles/datasets, Famous universities' websites, Environmental Monitoring Report, Statistical Yearbook, Diva GIS, IGBP, ClinVar, Copy Number Variations in Disease, Field trips	Correlation coefficients (30), Logistic regression (25), Generalized linear model (24), Multiple linear regression (9), Geographically weighted regression (9), Poisson regression (6), Geo-detector (5), ANOVA (5), PCA (5)	Administrative divisions (commune, district, city, province, country, etc.), Grids	See next row	Natural conditions mainly influence pathogen reproduction and survival, vector breeding and activity; socio-environmental factors mainly influence host (human) distribution and probability of contact between host and vector/pathogen; personal habits mainly influence host susceptibility; genetic inheritance and mutations mainly influence pathogen infectivity, hazard and host susceptibility, etc.
		Methods (*)	**Spatiotemporal scale**	**Factors (**+**)**	
Prediction and simulation (RD_3, data source as above)		Bayesian models (64), Compartment models (46), Network models (44), Phylogenetic models (24), Diffusion models (12), Linear regression model (11), Maximum entropy niche model (9), Gravity model (6), Agent-based spatio-temporal model (5), Cellular automata (5), Kriging interpolation (5), Radiation mode (5)	Administrative divisions (commune, district, city, province, country, etc.), Grids	Climate (115), Population (82), Human mobility (75), Variation (34), Vegetation (32), Heredity (31), Economy (28), Exposure to the infection (26), Land use (25), Hydrology (24), Topography (20), Urbanization (17), Transport (16), Vaccines (12), Medical level (9), Sunshine (8), Personal habits (5), Wind speed (5), Environmental pollution (5)	

“^*^”Means the number of publications using the method in the retrieved publications. “+”Means the number of publications considering the factor as an important factor affecting the formation of spatiotemporal patterns of infectious disease prevalence in the retrieved publications.

Common data sources, influencing factors, methods, spatial scales, and other characteristics were sorted for each research direction by content analysis (Table 3). There were strong relationships between the three research directions. After identifying the spatiotemporal patterns (RD_1) of infectious disease epidemics based on various methods, such as spatiotemporal scan, Moran' I, time series analysis (e.g., wavelet analysis), Getis-Ord Gi^*^, and kernel density, the influencing factors, including natural and social environments, individual habits, and microscopic molecular factors, were identified by correlation coefficients, logistic regression, various regression models and so on (RD_2). Based on their inferred mechanisms of action (influence processes), epidemic process simulation, epidemic risk prediction, and assessment of the effectiveness of prevention and control measures were done based on the detected influencing factors in which models, such as Bayesian models, compartment models (e.g., SEIR model), network models, phylogenetic models, niche models were employed (RD_3).

**Table 3:** Top 10 number of publications and collaboration.

**Grade of activity**	**Countries**	**Number as co-authors (perc)**	**Number as lead author (perc a, perc b)**	**Cooperation intensity**	**Top 3 countries with the most cooperation (Number)**
Grade 1	USA	338 (39.72%)	253 (74.85%, 29.73%)	272	England (36), China (32), France (18)
	China	180 (21.15%)	177 (98.33%, 20.80%)	108	US (32), Australia (14), England (13)
	England	104 (12.22%)	57 (54.81%, 6.70%)	150	US (36), China (13), France (12)
Grade 2	France	85 (9.99%)	59 (69.41%, 6.93%)	95	US, England, Belgium
	Australia	55 (6.46%)	37 (67.27%, 4.35%)	54	China, USA, England
	Brazil	53 (6.23%)	42 (79.25%, 4.94%)	36	USA, France, Portugal
	Canada	50 (5.88%)	35 (70.00%, 4.11%)	44	USA, China, England
Grade 3	Italy	36 (4.23%)	19 (52.78%, 2.23%)	42	USA, France, Switzerland
	Germany	33 (3.88%)	22 (66.67%, 2.59%)	39	USA, England, France
	Spain	30 (3.53%)	21 (70.00%, 2.47%)	35	USA, England, France

Lead author refers to the first or second author of the publication. Perc a refers to the number of publications in which the country is the lead author as a percentage of the number of publications in which the country is the co-author. Perc b refers to the number of publications in which the country is the lead author as a percentage of all publications in the field. Cooperation intensity refers to the number of collaborations with other countries.

### 3.4. Academic cooperation

Three grades of activity were divided based on the number of publications involved (NoPI) among the 10 countries with the highest NoPI (Table 3). Three countries (USA, China, and England) were in grade 1 (NoPI ≥ 100), four countries (France, Australia, Brazil, and Canada) were in grade 2 (50 ≤ NoPI < 100), and three countries (Italy, Germany, and Spain) were in grade 3 (30 ≤ NoPI < 50). The USA (338 participants, 253 leaders), China (180 participants, 177 leaders), and England (104 participants, 57 leaders) were the most active countries, both from the perspective of participants (as co-authors) and leaders (first and second authors of the publication). Specifically, 98% of the publications with Chinese scholars as co-authors had Chinese scholars as lead authors, indicating that Chinese scholars played a major role in the studies.

**Figure 4:**
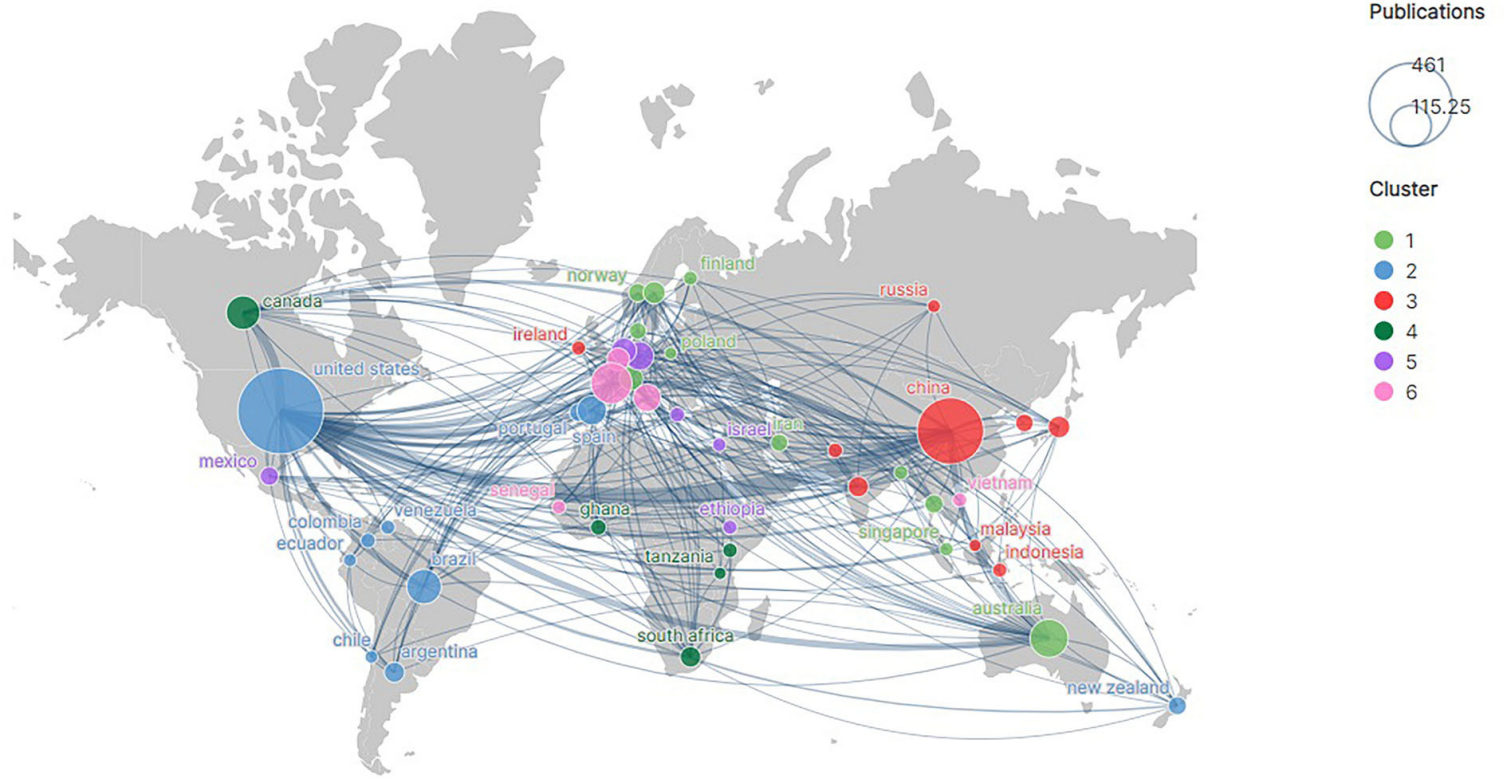
Country cooperation network. The thicker the line the greater the number of times the two countries work together. The size of the circle means the number of publications in the country.

The grade1 countries remained on the top with the highest intensity of international cooperation. Of all the cooperative relationships, the USA and England worked extremely closely (36 collaborations), followed by China and the USA (32 collaborations). Thus, it is evident that relatively active countries had a relatively high intensity of cooperation with other countries (Figure 4). This could be one of the reasons why the USA, China, and England were the three most active countries in this field.

### 3.5. Academic leadership

Three grades of leadership were divided based on the h-index of the publications (HoP) in the 10 countries with the highest HoP (Table 4). In this, one country (USA) was in grade 1 (HoP ≥ 50), four countries (England, China, France, and Canada) were in grade 2 (20 ≤ HoP < 50), and five countries (Australia, Italy, Germany, Brazil, and Spain) were in grade 3 (10 ≤ HoP < 20). This indicated that the developed countries had more academic leadership, although certain developing countries, such as China and Brazil, produced a large number of publications (Table 4).

**Table 4:** Top 10 countries with the most leadership.

**Grade of leadership**	**Countries**	**Cited frequency**	**Cited frequency per publication**	**h-index**
Grade 1	USA	21,581	63.85	60
Grade 2	England	9,663	92.91	39
	China	8,385	46.58	27
	France	3,232	38.02	26
	Canada	2,015	40.3	20
Grade 3	Australia	1,338	24.33	19
	Italy	1,810	50.28	16
	Germany	1,246	37.76	16
	Brazil	609	11.49	15
	Spain	755	25.17	13

The h-index means that at most N publications have been cited at least N times each.

Countries with high leadership had one or more relatively active (high number of publications) institutions (Table 5). The top 10 organizations listed in Table 5 were from the USA (4), France (3), China (2), and England (1), which were the top four countries in terms of leadership and the number of publications. However, two institutions from China (CDC and CAS) had the lowest leadership (h-index ≤ 14) among the ten most active institutions, yet they had a high number of publications.

**Table 5:** Top 10 institutions with the most publications.

**Ranking**	**Institution name**	**Country**	**Publications (percentage, %)**	**h-index**
1	UDICE-French Research Universities	France	44 (5.2)	19
2	University of California System	USA	40 (4.7)	20
3	Center national de la recherche scientifique (CNRS)	France	35 (4.1)	18
4	Chinese Center for Disease Control and Prevention (CDC)	China	31 (3.6)	14
5	Chinese Academy of Sciences (CAS)	China	30 (3.5)	13
6	University of London (UOL)	England	27 (3.2)	17
7	National Institutes of Health (NIH) - USA	USA	25 (2.9)	20
8	Institute of Research for Development (IRD)	France	23 (2.7)	14
9	NIH Fogarty International Center (FIC)	USA	23 (2.7)	19
10	Harvard University (Harvard)	USA	22 (2.7)	16

The h-index means that at most N publications have been cited at least N times each.

Countries with high academic leadership may have one or more highly cited author(s) in the country. In addition to WHO (232 cited) and Kulldorff (202 cited), the eight high-impact authors were from England (four) and the USA (four), the two countries with the highest leadership. However, Chinese scholars, who had a high number of publications (grade 1), did not make it to the list. These authors were frequently cited (Table 6), probably because the data, ideas and methods in their works discussed and solved key problems. WHO, the most frequently cited organization, provides important data for the field. It is the largest international inter-governmental health organization that hosts international epidemiological and health statistics operations, develops international names for diseases, causes of death, and public health implementation, and sets the standards for the international norms for diagnostic methods. Methodologically, Kulldorff's spatiotemporal scans and Anselin's Local Moran's I were common in this field and were often applied to identify dormant hotspots of the prevalence of infectious diseases. Ideologically, these authoritative scholars have made important explorations of RD_1 (e.g., Viboud, C, MADDEN, LV), RD_2 (e.g., ANDERSON, RM, Wesolowski, Amy), and RD_3 (e.g., Grenfell, BT, Ferguson, Neil M).

**Table 6:** Top 10 high-impact authors.

**Author**	**Frequently cited reasons**	**Frequency of cited**	**Country**
WHO (38–41)	Authoritative organizations, data statistics	232	United Nations
Kulldorff, M (42)	Method	204	Sweden
ANDERSON, RM (43)	Perspectives	106	England
Keeling, MJ (44)	Perspectives	83	England
MADDEN, LV (45)	Perspectives	78	USA
Grenfell, BT (46)	Perspectives	74	England
Wesolowski, Amy	Perspectives	74	USA
ANSELIN, L (47)	Method	67	USA
Ferguson, Neil M (48)	Perspectives	64	England
Viboud, C (49)	Perspectives	64	USA

Cited frequency refers to the cited frequency of the authors in the 851 publications retrieved.

**Figure 5:**
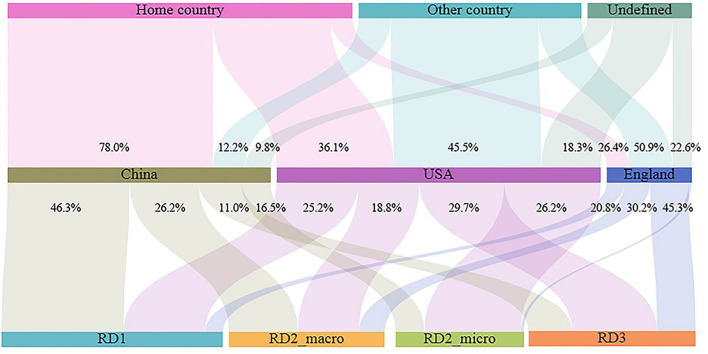
Research regions and research directions in three top active countries. The length of the bars means the number of publications. The percentage figure means the number of publications in the research directions or research area as a proportion of the total number of publications in the country. The statistics were based on research areas and research directions in publications where the country was the lead author (first or second author). Home country means the home country as the study area, while other country means other countries as the study area. RD2_macro means studies that detected the influence of macro factors on the epidemiology of infectious diseases, while RD2_micro means studies that detected the words and actions of micro factors on the epidemiology of infectious diseases.

There were clear differences in the research focus of the three countries (the USA, England, and China), despite having the highest leadership and number of publications (Figure 5). In general, studies by Chinese scholars have been biased toward the statistics and description of the spatiotemporal prevalence patterns of the infectious diseases (RD_1) and the analysis of the influence of macro (various social and environmental factors) factors on the prevalence patterns of the infectious diseases (RD_2; macro). However, scholars from the USA and England have produced more publications that Chinese scholars regarding the direction of the causes of infectious disease epidemics at the micro-scale (virus/bacterial genetics and mutation) (RD_2; micro) and the simulated dynamics of infectious disease transmissions (RD_3).

## 4. Discussion

This study identified the number of publications and citations, the spectrum of infectious diseases, major research directions and data-methodological-theoretical characteristics, academic collaborations, and leadership characteristics over the past 30 years in the mining of spatiotemporal patterns of infectious disease prevalence. The findings provide useful insights for advancing the detection of future spatiotemporal patterns of infectious diseases, the analysis of the influencing factors and driving mechanisms, academic cooperation, and prevention and control synergy.

The ever-increasing number of publications is likely related to a series of pandemics. The wide spread of SARS in 2003 (52) changed the field from a slow-rising phase to a fast-rising phase. Three extreme points observed in the fast-rising phase (2010, 2014, and 2017) corresponded with influenza (a pandemic transmitted from Mexico) (53), worldwide outbreaks of dengue (54), cholera epidemics of 2010 (55), unprecedented Ebola epidemics in West Africa (56), the most serious outbreak of dengue in China (57) in 2014, the cholera epidemic with thousands of infections in Yemen (58), the plague in Madagascar with more than 2,600 cases (59), and the 2017 influenza prevalence in the Northern Hemisphere (largest prevalence in the past decade) (59). The period of 2019-2021 (rapid rising phase), for the current world, was marked by the ongoing COVID-19 pandemic (60, 61), dengue fever epidemics in the subtropical and tropical regions (38), and the influenza pandemic worldwide (62). In summary, the characteristics of the infectious disease spectrum and its changes were, to some extent, strongly related to the pandemic or epidemic severity of major infectious diseases. Therefore, as the spectrum of infectious diseases illustrated (Figure 3), despite the remarkable achievements made by mankind to fight infectious diseases, such as SARS (63), cholera (64), plague (65), and avian influenza (66), the number of publications are likely to continue to rise since new infectious diseases (e.g., COVID-19) are emerging and pre-existing diseases (e.g., dengue and influenza) remain rampant.

This study uncovered the paradigmatic features of research in this field, including the logical nature of the three research directions and data-methods-theories/ideas. In spatiotemporal pattern detection (RD_1), wavelet analysis (67) was applied because it could detect periodic patterns of infectious disease epidemics and their changes over time. Spatiotemporal scanning (68) was widely employed owing to its ability to simultaneously detect specific clustering time intervals and spatial ranges. Co-location pattern mining algorithms (69) detected urban sites with a significant spatial correlation of infectious disease with case locations at the point scale. This method holds promise, however, there has been little research regarding it. RD_2 was used to identify the factors influencing the formation of the spatiotemporal patterns detected in RD_1, where the geo-detector (70) method, proposed in 2010, has become widely employed to simultaneously consider factors individually and interactively. With the improvement and perfection of machine learning and deep learning methods, methodologies, such as Bayesian models and Maxent (71), have often been applied to predict future epidemic risks of infectious diseases (RD_3) based on the influencing factors identified in RD_2. These can overcome the multi-collinearity between independent variables and prevent overfitting. In particular, they can detect the contribution of each variable while predicting the risk, and could become one of the dominant methods in this research direction. As can be seen, the three research directions were logically rigorous, which supported the framework of the research paradigm. However, the theories and methods involved in each direction are iterated and updated with time, indicating that the field deserves and requires in-depth research.

The spread of infectious diseases has no national boundaries and international cooperation is the only way and, the inevitable result of, fighting infectious diseases (25, 72). Over the last 30 years, developed countries have had a higher intensity of cross-country collaboration than developing countries, with more publications, h-index (citation), well-known relevant institutions, and scholars. These reflect stronger activity and leadership, which could be related to the prevalence of certain infectious diseases (52–56, 58, 59, 73). There has been a long-term accumulation of scientific research on the prevalence and control of infectious diseases in developed countries and a higher strategic profile (21, 62, 74–83). It was encouraging to note that China (a developing country) had a promising future in terms of international impact with a rapidly growing and leading level of intensity in international cooperation, publications, and citations, although its leadership was temporarily inferior to the USA and England. This could be related to the risk of traditional infectious diseases with long epidemiological history (84–88) and the increasing risk of importation of epidemics with frequent foreign exchanges (89) that requires China to make continued efforts in disease surveillance, data acquisition and sharing platform construction, and the development of universal methods or models (90, 91). However, China has a shortfall in terms of research areas of interest (only 13.5% of the publications focused on other countries) and research focus (smaller numbers of micro-level related research) compared to the USA and England. Thus, developing countries, such as China, need to expand the breadth and depth of their research to gain a broader, more in-depth, and stronger role in these collaborations (92, 93).

Although remarkable progress has been achieved, several points remain ill-considered and unresolved.

The spread, prevalence, and control of infectious diseases are complex ecological-geographical processes and the scale of effect (including time and space) is an important feature of this process because of the scale dependence on geographical objects (94, 95). Therefore, scale consistency is the primary issue that needs to be carefully considered when translating research findings into implementable measures. This has often been overlooked in existing research, although researchers chose the appropriate spatiotemporal scales before conducting a study. This study considered the spatial scale consistency by using a regular grid as a scale for conducting research by upscaling (fine-scale to coarse scale) and making recommendations. Regular grids can avoid the statistical and analytical issues that are likely to be caused by the research units assigned by the administrative divisions (districts, towns, streets, and villages) in relevant studies due to irregular and changeable shapes (96). In particular, these spatial grids have been gradually considered as the final units where prevention and control measures can produce practical effects in urban regions (89, 97). The appropriate grid scale can be chosen by comparing the ability (as reflected by some indicators, such as Moran's I) of a range of regular grids at different scales to characterize the spatiotemporal characteristics of infectious disease epidemics (51). However, if the resolution of the raw data is limited (e.g., at the county scale), when translating the findings into control measures at a finer scale (e.g., at the township scale), it must be carefully investigated if the findings had similar characteristics at a finer scale. It is important to note that the more the scale of the study differed from the scale of the proposed control measures, the results would be less credible.

Human mobility plays an important role in the transmission of infectious diseases (23, 98, 99), but most of these studies have focused only on infectious diseases that can be transmitted from human to human (e.g., COVID-19). Infectious illnesses include human-to-human (direct), human-vector (animal/insect)-human (indirect) types, etc. (1, 100). Human-to-human infectious diseases spread more rapidly and latently (e.g., COVID-19), in which human behavior factors have attracted more attention because of their direct impacts on the disease transmission and prevalence (23, 72, 101). In comparison, for human-vector (animal/insect)-human infectious diseases, the vector factors rather than human behavior factors were mainly concentrated in current studies although human behavior factors may pose impacts on the disease transmission and prevalence (102, 103). Actually, human often possesses higher mobility (an aspect of human behavior) than that of vectors (insects or animals), by which some infectious diseases (e.g., dengue, malaria, etc.,) transmitted by mosquitoes may be heavily affected by human behavior factors, especially when he/she has been infected or is a pathogen carrier (99, 104). The challenges of quantifying the impact of human mobility on insect- vector diseases at a fine spatial scale are related to the availability of data and the fusion of data from multiple sources (human mobility and infectious disease epidemics). With the advent of big data, data availability can be expected related to population location heat maps, bus/subway smart card swipe data, and taxi track data (105, 106). For fusing data from multiple sources, this study suggests clustering, random forest, and deep learning (e.g., convolution neural network) to convert human mobility data with fine time granularity into patterns at the same temporal scale as the incidence rate. Hence, the association between human mobility patterns (category variables) and the prevalence of infectious diseases can be detected at fine spatial scales using methods such as geo-detectors. Thus, human mobility characteristics can be further explained based on the point of interest (POI) and land use types of different mobility patterns to infer the processes and causes of infectious disease epidemics.

This study had certain limitations. The data were obtained only from the WoS core database, which resulted in some relevant articles being excluded. In the future, the authors could consider integrating data from multiple databases, such as Scopus and PubMed. In addition, we searched for publications based on the existence of specific keywords in the title, abstract, and keywords of the article and may have missed some relevant publications. Additional information (e.g., conclusions) must be combined to search the publications.

## 5. Conclusion

This study revealed that the epidemiological and control situation of infectious diseases over the past 30 years has led to an increase in the publication of spatiotemporal pattern mining studies and a change in the spectrum of focused diseases. Developed countries have stronger leadership than developing countries in this field. Existing research, on the whole, has followed the logic and paradigm of pattern-factors-mechanisms. The authors suggest that international cooperation must continue to advance and developing countries, such as China, must strengthen their breadth and depth of research to enhance their influence in the field. Scholars need to strengthen the exploration of scale effects and human mobility in the field. Thus, this study provides useful insights for scholars and relevant health authorities to understand the progress of existing research and the remaining gaps, and develop relevant research, prevention, and control strategies.

## Author contributions

WL: conceptualization, methodology, visualization, validation, writing–original draft, supervision, and writing–review and editing. HR: data curation, investigation, formal analysis, writing–original draft, and writing–review and editing. All authors contributed to the article and approved the submitted version.

## References

[1] Agany DDM Pietri JE Gnimpieba EZ. Assessment of vector-host-pathogen relationships using data mining and machine learning. Comput Struct Biotechnol J. (2020) 18:1704–21. 10.1016/j.csbj.2020.06.031 32670510 PMC7340972

[2] Bloom DE Black S Rappuoli R. Emerging infectious diseases: a proactive approach. Proc Nat Acad Sci. (2017) 114:4055. 10.1073/pnas.1701410114 28396438 PMC5402424

[3] Salje H Lessler J Maljkovic Berry I Melendrez Melanie C Endy T Kalayanarooj S. Dengue diversity across spatial and temporal scales: local structure and the effect of host population size. Science. (2017) 355:1302–6. 10.1126/science.aaj9384 28336667 PMC5777672

[4] Dayou L Huiling C Hong Q Bo Y. Advances in spatiotemporal data mining. J Comput Res Dev. (2013) 225–39. 10.11867/j.issn.1001-8166.2011.04.0449

[5] Tao P Yaxi L Sihui G Hua S Yunyan D Ting M. Principle of big geodata mining. Acta Geogr Sin. (2019) 74:586–98. 10.11821/dlxb201903014

[6] Bin Z Jinlin L Ying M. Spatial correlation of incidence rate of typical notifiable infectious diseases in China. China J Publ Health. (2018) 34:4–8. 10.11847/zgggws1114291

[7] Kuo TM Meyer AM Baggett CD Olshan AF. Examining determinants of geographic variation in colorectal cancer mortality in North Carolina: a spatial analysis approach. Cancer Epidemiol. (2019) 59:8–14. 10.1016/j.canep.2019.01.002 30640041

[8] Ord JK Getis A. Local spatial autocorrelation statistics: distributional issues and an application. Geogr Anal. (2010) 27:286–306. 10.1111/j.1538-4632.1995.tb00912.x

[9] Kanji JN Isaac A Gregson D Mierzejewski M Shpeley D Tomlin P. Epidemiology of ticks submitted from human hosts in Alberta, Canada (2000-2019). Emerg Microbes Infect. (2022) 11:284–92. 10.1080/22221751.2022.2027217 34991433 PMC8812759

[10] Nasirpour MH Sharifi A Ahmadi M Ghoushchi SJ. Revealing the relationship between solar activity and COVID-19 and forecasting of possible future viruses using multi-step autoregression (MSAR). Environ Sci Pollut Res. (2021) 28:38074–84. 10.1007/s11356-021-13249-2 33725302 PMC7961325

[11] Cao Z Liu T Li X Wang J Lin HL Chen LL. Individual and interactive effects of socio-ecological factors on dengue fever at fine spatial scale: a geographical detector-based analysis. Int J Environ Res Public Health. (2017) 14:14. 10.3390/ijerph14070795 28714925 PMC5551233

[12] Zheng L Ren HY Shi RH Lu L. Spatiotemporal characteristics and primary influencing factors of typical dengue fever epidemics in China. Infect Dis Poverty. (2019) 8:12. 10.1186/s40249-019-0533-9 30922405 PMC6440137

[13] Ren H Wu W Li T Yang Z. Urban villages as transfer stations for dengue fever epidemic: a case study in the Guangzhou, China. PLoS Neglect Trop Dis. (2019) 13:e0007350. 10.1371/journal.pntd.0007350 31022198 PMC6504109

[14] Rasam AA Shariff NM Dony J Misni A. Socio-environmental factors and tuberculosis: an exploratory spatial analysis in Peninsular Malaysia. Int J Eng Technol. (2018) 7:187–92. 10.14419/ijet.v7i3.11.15958

[15] Ge E Lai PC Zhang X Yang X Li X Wang H. Regional transport and its association with tuberculosis in the Shandong province of China, 2009–2011. J Trans Geogr. (2015) 46:232–43. 10.1016/j.jtrangeo.2015.06.021

[16] Krieger MS Denison CE Anderson TL Nowak MA Hill AL. Population structure across scales facilitates coexistence and spatial heterogeneity of antibiotic-resistant infections. PLoS Comput Biol. (2020) 16:34. 10.1371/journal.pcbi.1008010 32628660 PMC7365476

[17] Nelson KN Shah NS Mathema B Ismail N Brust JCM Brown TS. Spatial patterns of extensively drug-resistant tuberculosis transmission in KwaZulu-Natal, South Africa. J Infect Dis. (2018) 218:1964–73. 10.1093/infdis/jiy394 29961879 PMC6217717

[18] Tang J Zhang SX Zhang J Li XY Zhou JF Zou SM. Profile and generation of reduced neuraminidase inhibitor susceptibility in highly pathogenic avian influenza H7N9 virus from human cases in Mainland of China, 2016-2019. Virology. (2020) 549:77–84. 10.1016/j.virol.2020.07.018 32853849

[19] Zhang YW Wang XF Li YF Ma JQ. spatiotemporal analysis of influenza in China, 2005-2018. Sci Rep. (2019) 9:12. 10.1038/s41598-019-56104-8 31873144 PMC6928232

[20] Nguyen LT Stevenson MA Firestone SM Sims LD Chu DH Nguyen LV. Spatiotemporal and risk analysis of H5 highly pathogenic avian influenza in Vietnam, 2014-2017. Prev Vet Med. (2020) 178:10. 10.1016/j.prevetmed.2019.04.007 31113666

[21] Finger F Genolet T Mari L de Magny GC Manga NM Rinaldo A. Mobile phone data highlights the role of mass gatherings in the spreading of cholera outbreaks. Proc Natl Acad Sci U S A. (2016) 113:6421–6. 10.1073/pnas.1522305113 27217564 PMC4988598

[22] Kraemer MUG Yang CH Gutierrez B Wu CH Klein B Pigott DM. The effect of human mobility and control measures on the COVID-19 epidemic in China. Science. (2020) 368:493-+. 10.1126/science.abb4218 32213647 PMC7146642

[23] Kraemer MUG Golding N Bisanzio D Bhatt S Pigott DM Ray SE. Utilizing general human movement models to predict the spread of emerging infectious diseases in resource poor settings. Sci Rep. (2019) 9:11. 10.1038/s41598-019-41192-3 30914669 PMC6435716

[24] Yang WT Zhang JT Ma RL. The prediction of infectious diseases: a bibliometric analysis. Int J Environ Res Public Health. (2020) 17:19. 10.3390/ijerph17176218 32867133 PMC7504049

[25] Sweileh WM. Bibliometric analysis of peer-reviewed literature on climate change and human health with an emphasis on infectious diseases. Global Health. (2020) 16:17. 10.1186/s12992-020-00576-1 32384901 PMC7206222

[26] dos Santos BS Steiner MTA Fenerich AT Lima RHP. Data mining and machine learning techniques applied to public health problems: a bibliometric analysis from 2009 to 2018. Comput Ind Eng. (2019) 138:11. 10.1016/j.cie.2019.106120

[27] Agnusdei GP Coluccia B. Sustainable agrifood supply chains: Bibliometric, network and content analyses. Sci Total Environ. (2022) 824:10. 10.1016/j.scitotenv.2022.153704 35134421

[28] Su YA Yu YN Zhang N. Carbon emissions and environmental management based on Big Data and Streaming Data: a bibliometric analysis. Sci Total Environ. (2020) 733:11. 10.1016/j.scitotenv.2020.138984 32446050

[29] Mongeon P Paul-Hus A. The journal coverage of Web of Science and Scopus: a comparative analysis. Scientometrics. (2016) 106:213–28. 10.1007/s11192-015-1765-5

[30] Zhu JW Liu WS. A tale of two databases: the use of Web of Science and Scopus in academic papers. Scientometrics. (2020) 123:321–35. 10.1007/s11192-020-03387-8

[31] Falagas ME Pitsouni EI Malietzis GA Pappas G. Comparison of pubmed, scopus, web of science, and google scholar: strengths and weaknesses. FASEB J. (2008) 22:338–42. 10.1096/fj.07-9492LSF 17884971

[32] Pan XL Yan EJ Cui M Hua WN. Examining the usage, citation, and diffusion patterns of bibliometric mapping software: a comparative study of three tools. J Informetr. (2018) 12:481–93. 10.1016/j.joi.2018.03.005

[33] Chen CM. CiteSpace II: Detecting and visualizing emerging trends and transient patterns in scientific literature. J Am Soc Inf Sci Technol. (2006) 57:359–77. 10.1002/asi.20317

[34] Chen CM Ibekwe-SanJuan F Hou JH. The structure and dynamics of cocitation clusters: a multiple-perspective cocitation analysis. J Am Soc Inf Sci Technol. (2010) 61:1386–409. 10.1002/asi.21309

[35] van Eck NJ Waltman L. Software survey: VOSviewer, a computer program for bibliometric mapping. Scientometrics. (2010) 84:523–38. 10.1007/s11192-009-0146-3 20585380 PMC2883932

[36] Jing F Jingda D. Comparison of Visualization Principles between Citespace and VOSviewer. J Lib Inf Sci Agric. (2019) 31:31–7. 10.13998/j.cnki.issn1002-1248.2019.10.19-0776

[37] Gaur A Kumar M. A systematic approach to conducting review studies: An assessment of content analysis in 25 years of IB research. J World Bus. (2018) 53:280–9. 10.1016/j.jwb.2017.11.003

[38] Biswal S Reynales H Saez-Llorens X Lopez P Borja-Tabora C Kosalaraksa P. Efficacy of a tetravalent dengue vaccine in healthy children and adolescents. N Engl J Med. (2019) 381:2009–19. 10.1056/NEJMoa1903869 31693803

[39] World Hlth O. Cholera vaccine: WHO position paper, August 2017-Recommendations. Vaccine. (2018) 36:3418–20. 10.1016/j.vaccine.2017.09.034 29555219

[40] Sweileh WM Moh'd Mansour A. Bibliometric analysis of global research output on antimicrobial resistance in the environment (2000-2019). Glob Health Res Policy. (2020) 5:37. 10.1186/s41256-020-00165-0 32775695 PMC7398083

[41] Abbafati C Abbas KM Abbasi M Abbasifard M Abbasi-Kangevari M Abbastabar H. Global burden of 369 diseases and injuries in 204 countries and territories, 1990-2019: a systematic analysis for the Global Burden of Disease Study 2019. Lancet. (2020) 396:1204–22. 10.1016/S0140-6736(20)30925-9 33069326 PMC7567026

[42] Kulldorff M. A spatial scan statistic. Commun Stat-Theory Methods. (1997) 26:1481–96. 10.1080/03610929708831995

[43] Anderson RM May RM. Population biology of infectious-diseases 1. Nature. (1979) 280:361–7. 10.1038/280361a0 460412

[44] Keeling MJ Eames KTD. Networks and epidemic models. J R Soc Interface. (2005) 2:295–307. 10.1098/rsif.2005.0051 16849187 PMC1578276

[45] Madden LV Hughes G. Plant-disease incidence. - distribution, heterogeneity, and temporal analysis. Annu Rev Phytopathol. (1995) 33:529–64. 10.1146/annurev.py.33.090195.002525 18999972

[46] Grenfell BT Pybus OG Gog JR Wood JLN Daly JM Mumford JA. Unifying the epidemiological and evolutionary dynamics of pathogens. Science. (2004) 303:327–32. 10.1126/science.1090727 14726583

[47] Anselin L. Local indicators of spatial association. - LISA. Geogr Anal. (1995) 27:93–115. 10.1111/j.1538-4632.1995.tb00338.x

[48] Ferguson NM Cummings DAT Fraser C Cajka JC Cooley PC Burke DS. Strategies for mitigating an influenza pandemic. Nature. (2006) 442:448–52. 10.1038/nature04795 16642006 PMC7095311

[49] Viboud C Bjornstad ON Smith DL Simonsen L Miller MA Grenfell BT. Synchrony, waves, and spatial hierarchies in the spread of influenza. Science. (2006) 312:447–51. 10.1126/science.1125237 16574822

[50] Thai KTD Cazelles B Nam VN Long TV Boni MF Farrar J. Dengue dynamics in binh thuan province, southern vietnam: periodicity, synchronicity and climate variability. PLoS Negl Trop Dis. (2010) 4:8. 10.1371/journal.pntd.0000747 20644621 PMC2903474

[51] Ren HY Lu WL Li XQ Shen HC. Specific urban units identified in tuberculosis epidemic using a geographical detector in Guangzhou, China. Infect Dis Poverty. (2022) 11:12. 10.1186/s40249-022-00967-z 35428318 PMC9012046

[52] Lipsitch M Cohen T Cooper B Robins JM Ma S James L. Transmission dynamics and control of severe acute respiratory syndrome. Science. (2003) 300:1966–70. 10.1126/science.1086616 12766207 PMC2760158

[53] Bautista E Chorpitayasunondh T Gao ZC Harper SA Shaw M Uyeki TM. Medical progress: clinical aspects of pandemic 2009 Influenza A (H1N1) virus infection. N Engl J Med. (2010) 362:1708–19. 10.1056/NEJMra1000449 20445182

[54] Lingxia Z Yongyi W Wen C Jun L Zhu W. Hot spots of infectious diseases occurring in 2010. Infect Dis Inf. (2011) 24:1–5. 10.3969/j.issn.1007-8134.2011.01.001

[55] Chin CS Sorenson J Harris JB Robins WP Charles RC Jean-Charles RR. The origin of the haitian cholera outbreak strain. N Engl J Med. (2011) 364:33–42. 10.1056/NEJMoa1012928 21142692 PMC3030187

[56] Aylward B Barboza P Bawo L Bertherat E Bilivogui P Blake I. Ebola virus disease in West Africa - the first 9 months of the epidemic and forward projections. N Engl J Med. (2014) 371:1481–95. 10.1056/NEJMoa1411100 25244186 PMC4235004

[57] Sang SW Gu SH Bi P Yang WZ Yang ZC Xu L. Predicting unprecedented dengue outbreak using imported cases and climatic factors in Guangzhou, 2014. Plos Neglect Trop Dis. (2015) 9:12. 10.1371/journal.pntd.0003808 26020627 PMC4447292

[58] Camacho A Bouhenia M Alyusfi R Alkohlani A Naji MAM de Radigues X. Cholera epidemic in Yemen, 2016-18: an analysis of surveillance data. Lancet Glob Health. (2018) 6:E680–E90. 10.1016/S2214-109X(18)30230-4 29731398 PMC5952990

[59] Shuo L hui ZY Yongyi Z Mei L Wen H Yangling Z. Hot spots review of global infectious diseases in 2017. Infect Dis Inf . (2018) 31:5–10. 10.3969/j.issn.1007-8134.2018.01.002

[60] Gao ZR Xu YH Sun C Wang X Guo Y Qiu S. A systematic review of asymptomatic infections with COVID-19. J Microbiol Immunol Infect. (2021) 54:12–6. 10.1016/j.jmii.2020.05.001 32425996 PMC7227597

[61] Miller IF Metcalf CJE. Assessing the risk of vaccine-driven virulence evolution in SARS-CoV-2. R Soc Open Sci. (2022) 9:16. 10.1098/rsos.211021 35070341 PMC8728167

[62] Paules C Subbarao K. Influenza. Lancet. (2017) 390:697–708. 10.1016/S0140-6736(17)30129-0 28302313

[63] Xu RH. Chance missed, but still there! Memoirs at the 10th anniversary of 2003 SARS outbreak. J Thorac Dis. (2013) 5:S90–S3. 10.3978/j.issn.2072-1439.2013.04.07 23977441 PMC3747526

[64] Yang WZ. Dramatic achievements in infectious disease prevention and treatment in China during the past 70 years. Zhonghua Liu Xing Bing Xue Za Zhi. (2019) 40:1493–8. 10.3760/cma.j.issn.0254-6450.2019.12.001 32062906

[65] Lianxu X. Achievements of plague prevention and control in China. Dis Surveill. (2021) 36:650–2. 10.3784/jbjc.202107260415

[66] Zhang Y Wang Y Wang F Wang J Bi Y Yin Y. Progress and achievements in the research on avian influenza in China. Microbiol China. (2014) 41:497−503. 10.13344/j.microbiol.china.130619

[67] Xinlou L. Spatiotemporal Distribution and Risk Assessment of Human Infections with Avian Influenza and Dengue Fever [博士]: Academy of Military Medical Sciences. (2015) 11. 10.7666/d.Y2993764

[68] Lawson A. Statistical methods for disease clustering. Ann Epidemiol. (2010) 20:964. 10.1016/j.annepidem.2010.07.101

[69] Huang Y Shekhar S Xiong H. Discovering colocation patterns from spatial data sets: a general approach. IEEE Trans Knowl Data Eng. (2004) 16:1472–85. 10.1109/TKDE.2004.90

[70] Wang JF Li XH Christakos G Liao YL Zhang T Gu X. Geographical detectors-based health risk assessment and its application in the neural tube defects study of the Heshun Region, China. Int J Geogr Inf Sci. (2010) 24:107–127. 10.1080/13658810802443457

[71] Arpaci A Malowerschnig B Sass O Vacik H. Using multi variate data mining techniques for estimating fire susceptibility of Tyrolean forests. Appl Geogr. (2014) 53:258–70. 10.1016/j.apgeog.2014.05.015

[72] Nalbandian A Sehgal K Gupta A Madhavan MV McGroder C Stevens JS. Post-acute COVID-19 syndrome. Nat Med. (2021) 27:601–15. 10.1038/s41591-021-01283-z 33753937 PMC8893149

[73] Guan W Ni Z Hu Y Liang W Ou C He J. Clinical characteristics of coronavirus disease 2019 in China. N Engl J Med. (2020) 382:1708–20. 10.1056/NEJMoa2002032 32109013 PMC7092819

[74] Githeko AK Lindsay SW Confalonieri UE Patz JA. Climate change and vector-borne diseases: a regional analysis. Bull World Health Organ. (2000) 78:1136–47. 10.1590/S0042-96862000000900009 11019462 PMC2560843

[75] Nash D Mostashari F Fine A Miller J O'Leary D Murray K. The outbreak of West Nile virus infection in the New York City area in 1999. N Engl J Med. (2001) 344:1807–14. 10.1056/NEJM200106143442401 11407341

[76] Rosenberg R Lindsey NP Fischer M Gregory CJ Hinckley AF Mead PS. Vital signs: trends in reported vectorborne disease cases - United States and territories, 2004-2016. MMWR. (2018) 67:496–501. 10.15585/mmwr.mm6717e1 29723166 PMC5933869

[77] Reif S Safley D McAllaster C Wilson E Whetten K. State of HIV in the US deep south. J Community Health. (2017) 42:844–53. 10.1007/s10900-017-0325-8 28247067

[78] Rolfes MA Flannery B Chung JR O'Halloran A Garg S Belongia EA. Effects of influenza vaccination in the United States during the 2017-2018 influenza season. Clin Infect Dis. (2019) 69:1845–53. 10.1093/cid/ciz075 30715278 PMC7188082

[79] Wang YD Yan ZH Wang D Yang MT Li ZQ Gong XR. Prediction and analysis of COVID-19 daily new cases and cumulative cases: times series forecasting and machine learning models. BMC Infect Dis. (2022) 22(Suppl 1):12. 10.1186/s12879-022-07472-6 35614387 PMC9131989

[80] Bhatt S Gething PW Brady OJ Messina JP Farlow AW Moyes CL. The global distribution and burden of dengue. Nature. (2013) 496:504–7. 10.1038/nature12060 23563266 PMC3651993

[81] Stoddard ST Forshey BM Morrison AC Paz-Soldan VA Vazquez-Prokopec GM Astete H. House-to-house human movement drives dengue virus transmission. Proc Natl Acad Sci U S A. (2013) 110:994–9. 10.1073/pnas.1213349110 23277539 PMC3549073

[82] Lemey P Rambaut A Drummond AJ Suchard MA. Bayesian phylogeography finds its roots. PLoS Comput Biol. (2009) 5:16. 10.1371/journal.pcbi.1000520 19779555 PMC2740835

[83] Kraemer MUG Sinka ME Duda KA Mylne AQN Shearer FM Barker CM. The global distribution of the arbovirus vectors Aedes aegypti and Ae. albopictus. eLife. (2015) 4:18. 10.7554/eLife.08347 26126267 PMC4493616

[84] Brody H Rip MR Vinten-Johansen P Paneth N Rachman S. Map-making and myth-making in Broad Street: the London cholera epidemic, 1854. Lancet. (2000) 356:64–8. 10.1016/S0140-6736(00)02442-9 10892779

[85] Wu X Nethery RC Sabath MB. Air pollution and COVID-19 mortality in the United States: Strengths and limitations of an ecological regression analysis [J]. Science Advances. (2020) 6:eabd4049. 10.1126/sciadv.abd4049 33148655 PMC7673673

[86] Olea RA Christakos G. Duration of urban mortality for the 14th-century black death epidemic. Hum Biol. (2005) 77:291–303. 10.1353/hub.2005.0051 16392633

[87] Dawood FS Jain S Finelli L Shaw MW Lindstrom S Garten RJ. Emergence of a novel swine-origin Influenza A (H1N1) virus in humans novel swine-origin Influenza A (H1N1) virus investigation team. N Engl J Med. (2009) 360:2605–15. 10.1056/NEJMoa0903810 19423869

[88] Xiong Y Chen Q. Epidemiology of dengue fever in China since 1978. Nan Fang Yi Ke Da Xue Xue Bao. (2014) 34:1822–5. 10.3969/j.issn.1673-4254.2014.12.24 25537911

[89] Yingyue L Zhaohui L. Review on the development of digital city management in China. Intell Build City Inf . (2017) 2:28–32. 10.13655/j.cnki.ibci.2017.02.004

[90] The World Bank. (2022). Available online at: https://wwwworldbankorg/en/home (accessed August 17, 2022).

[91] Huang C Hai-Xia MA Liang HG. Global countermeasures against Ebola virus disease and enlightenment on preventing and controlling fulminating infectious diseases in China. Military Medical Sciences (2018) 42:786–91.

[92] Lei ZM. Historical study of the outside planting substitution policy of communist party of china in the last twenty years-taking planting substitution assistance to myanmar and laos by agricultural bureau of xishuang banna as an example. In: 6th International Conference on Social Science, Education and Humanities Research (SSEHR). Jinan, Peoples R China. Paris: Atlantis Press. (2017).

[93] Peilong Liu A. China's distinctive engagement in global health. Lancet. (2014) 384:793–804. 10.1016/S0140-6736(14)60725-X 25176550 PMC7159291

[94] Shuangcheng L Yunlong C. Some scaling issues of geography. Geogr Res. (2005) 1:11–8. 10.3969/j.issn.1007-6301.2004.02.003

[95] Mingliang L Xianming T Jiyuan L Dafang Z. Research on scaling effect based on 1 km grid cell data. Natl Remote Sens Bull. (2001) 183–90+243–4. 10.3321/j.issn:1007-4619.2001.03.005

[96] Hongyan R Duanyang X Xiaoming S Jianwei X Dafang Z Gonghuan Y. Characterisation of gastric cancer and its relation to environmental factors: a case study in Shenqiu County, China. Int J Environ Health Res. (2016) 26:1–10. 10.1080/09603123.2014.1003040 25608493

[97] Yaojun Y. On Grid Management in City Management. Urban Problems. (2006). 2:76–9. 10.3969/j.issn.1002-2031.2006.02.017

[98] Masui H Kakitani I Ujiyama S Hashidate K Shiono M Kudo K. Assessing potential countermeasures against the dengue epidemic in non-tropical urban cities. Theor Biol Med Model. (2016) 13:13. 10.1186/s12976-016-0039-0 27072122 PMC4828873

[99] Wen TH Hsu CS Hu MC. Evaluating neighborhood structures for modeling intercity diffusion of large-scale dengue epidemics. Int J Health Geogr. (2018) 17:15. 10.1186/s12942-018-0131-2 29724243 PMC5934834

[100] Chae S Kwon S Lee D. Predicting infectious disease using deep learning and big data. Int J Environ Res Publ Health. (2018) 15:1596. 10.3390/ijerph15081596 30060525 PMC6121625

[101] Zhang Y Li YB Yang B Zheng X Chen M. Risk assessment of COVID-19 based on multisource data from a geographical viewpoint. IEEE Access. (2020) 8:125702–13. 10.1109/ACCESS.2020.3004933

[102] Stoddard ST Morrison AC Vazquez-Prokopec GM Soldan VP Kochel TJ Kitron U. The role of human movement in the transmission of vector-borne pathogens. Plos Neglect Trop Dis. (2009) 3:9. 10.1371/journal.pntd.0000481 19621090 PMC2710008

[103] Zhu GH Liu T Xiao JP Zhang B Song T Zhang YH. Effects of human mobility, temperature and mosquito control on the spatiotemporal transmission of dengue. Sci Total Environ. (2019) 651:969–78. 10.1016/j.scitotenv.2018.09.182 30360290

[104] Falcon-Lezama JA Martinez-Vega RA Kuri-Morales PA Ramos-Castaneda J Adams B. Day-to-day population movement and the management of dengue epidemics. Bull Math Biol. (2016) 78:2011–33. 10.1007/s11538-016-0209-6 27704330 PMC5069346

[105] Xiping Y Zhixiang F. Recent progress in studying human mobility and urban spatial structure based on mobile location big data. Prog Geog. (2018) 37:880–9. 10.18306/dlkxjz.2018.07.002

[106] Gonzalez MC Hidalgo CA Barabasi AL. Understanding individual human mobility patterns. Nature. (2008) 453:779–82. 10.1038/nature06958 18528393

