# Supplementary material for: Diseases spectrum in the field of spatiotemporal patterns mining of infectious diseases epidemics: A bibliometric and content analysis
Source: Front Public Health. 2023 Jan 9;10:1089418. doi: 10.3389/fpubh.2022.1089418 (PMC9868952; doi:10.3389/fpubh.2022.1089418)
Supplement: Supplementary file 1 [file Data_Sheet_1.docx]

Table S1 Research theme information table

| Theme No. | Research Content | Top 5 Topic Words |
| --- | --- | --- |
| #0 | Exploring the spatiotemporal distribution and dynamic characteristics of **dengue fever** prevalence; detecting the influence of population mobility, etc. on the spread of dengue fever; explaining the spatiotemporal patterns of dengue fever prevalence from the perspective of spatial differentiation of the pathogen (dengue virus) | human infectious diseases; spatiotemporal variation; google location data; multi-patch departmental model; different spatial scale |
| #1 | Mining spatiotemporal aggregation characteristics of **COVID-19** prevalence; Detecting the influence of human mobility and other factors on COVID-19 prevalence; Emulating of spatiotemporal patterns of future COVID-19 prevalence; Evaluating the effects of measures | covid-19 pandemic; clustering radius; population mobility; mainland China; New York State |
| #2 | Molecular epidemiological studies of **influenza** viruses, including the spatiotemporal differentiation and stochasticity of influenza virus evolution, and its impact on influenza prevalence patterns; evaluating the effectiveness of various interventions for influenza prevention and control | viral genetic variation; viral phylogenies; activity-based model; bird flu; molecular epidemiology |
| #4 | Detecting spatiotemporal aggregation patterns of **avian influenza** prevalence; analyzing its influencing factors; spatiotemporal spread/dynamic simulation; future risk mapping; molecular epidemiological study of avian influenza viruses | wild bird; h5n1 virus; pathogenic avian influenza; frequent mixed infection; h5n1 outbreak |
| #5 | The importance of **human mobility** on the mining and simulation of spatiotemporal patterns of infectious diseases prevalence; data/theories/methods commonly used to quantify population mobility | mobile phone data; Ebola virus disease; mobile phone; future epidemics; mobile phone record |
| #7 | Influence of precipitation and other climatic conditions, water-environment systems, and population mobility on **cholera** outbreaks; prediction of spatiotemporal patterns of cholera prevalence and assessment of the effects of epidemic interventions; influence of precipitation, temperature and other climatic conditions, and human mobility on **malaria** prevalence | malaria incidence; water system; pathogen invasion; epidemic cholera; dominant eigenvector |
| #8 | Excavation of periodic patterns and spatiotemporal aggregation patterns of **HFMD** epidemics; application of time series analysis methods to detect seasonal characteristics and the influence of climatic factors on its prevalence | hfmd incidence; Vietnam; mouth disease; southeast Asia; meteorological factor |
| #9 | Application of **network theory** to spatiotemporal pattern mining of infectious disease prevalence | pathogen spread; plant trade network; plant diseases; network theory; network epidemiology |
| #11 | Analysis of spatiotemporal aggregation patterns and spatiotemporal heterogeneity characteristics of mosquito-borne infectious diseases such as **dengue fever**; analysis of the influence of environmental socio-economic risk factors | dengue incidence; children; high-risk village; dengue; Cirebon |

Note: Clusters included with a publication count of 10 or less were omitted, which resulted in discontinuous cluster numbers.


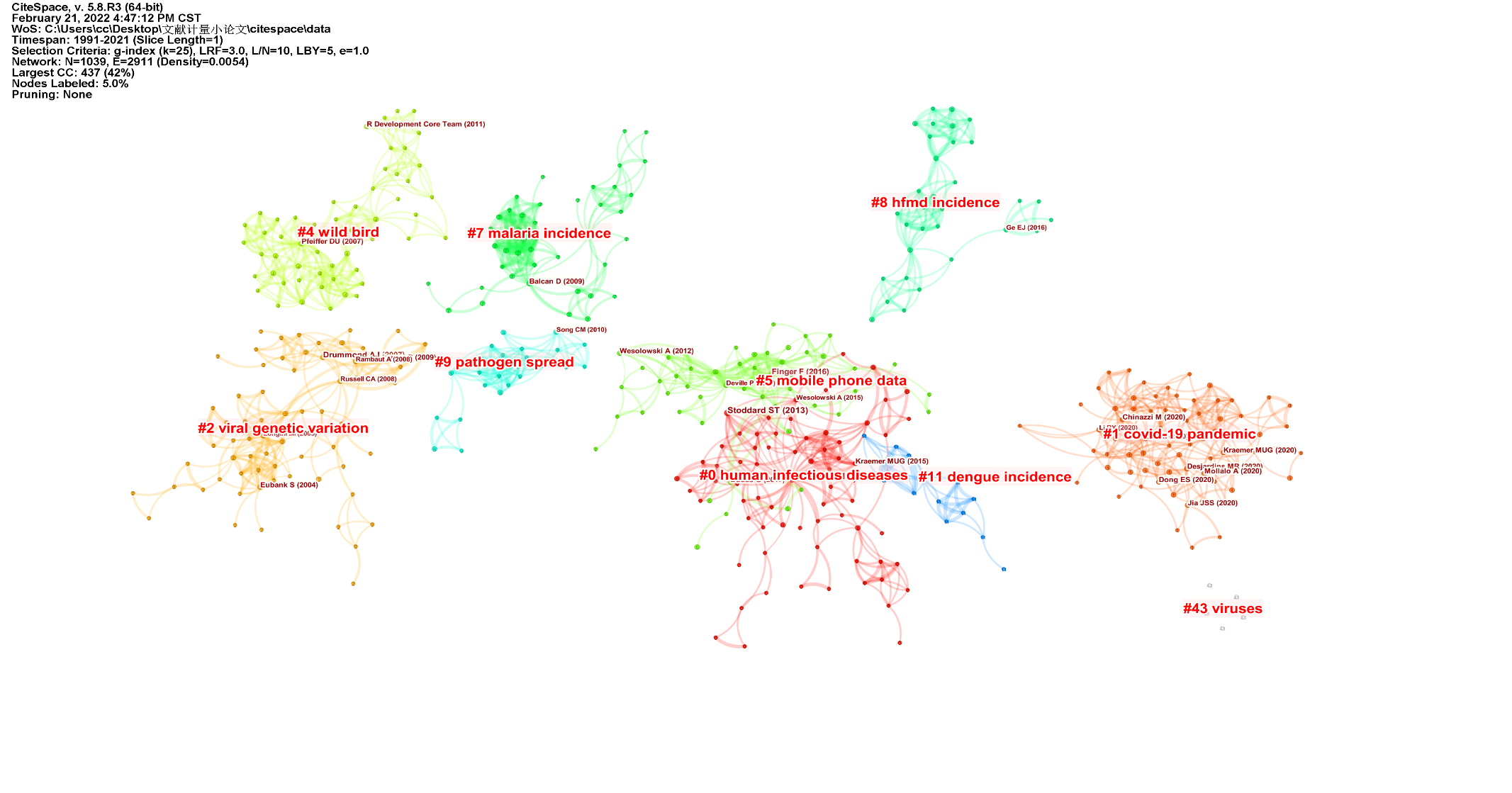


Fig. S1 Topic clustering by CiteSpace5.8.R3
